# Extensive polyostotic fibrous dysplasia evaluated for malignant transformation with ^99m^Tc-MDP bone scan and ^18^F-FDG PET/CT

**DOI:** 10.1259/bjrcr.20150440

**Published:** 2016-07-28

**Authors:** William Makis, Stephan Probst

**Affiliations:** ^1^Department of Diagnostic Imaging, Cross Cancer Institute, Edmonton, AB, Canada; ^2^Department of Nuclear Medicine, Jewish General Hospital, McGill University, Montreal, QC, Canada

## Abstract

Fibrous dysplasia accounts for approximately 7% of benign bone tumours and is a developmental disorder of unknown aetiology. Malignant transformation has been reported in 0.4% of all cases of fibrous dysplasia, and the use of ^18^F-fludeoxyglucose positron emission tomography/CT scan in the evaluation of malignant transformation has not yet been established. A 72-year-old male with a long-standing history of polyostotic fibrous dysplasia presented with chest and back pain and was evaluated with a ^99m^Tc-methylene diphosphonate bone scan as well as an ^18^F-fludeoxyglucose positron emission tomography/CT scan to define the extent of bone involvement and assess for possible malignant transformation. We present the imaging findings as well as the long-term follow-up of this case.

## Case report

**Figure 1.**
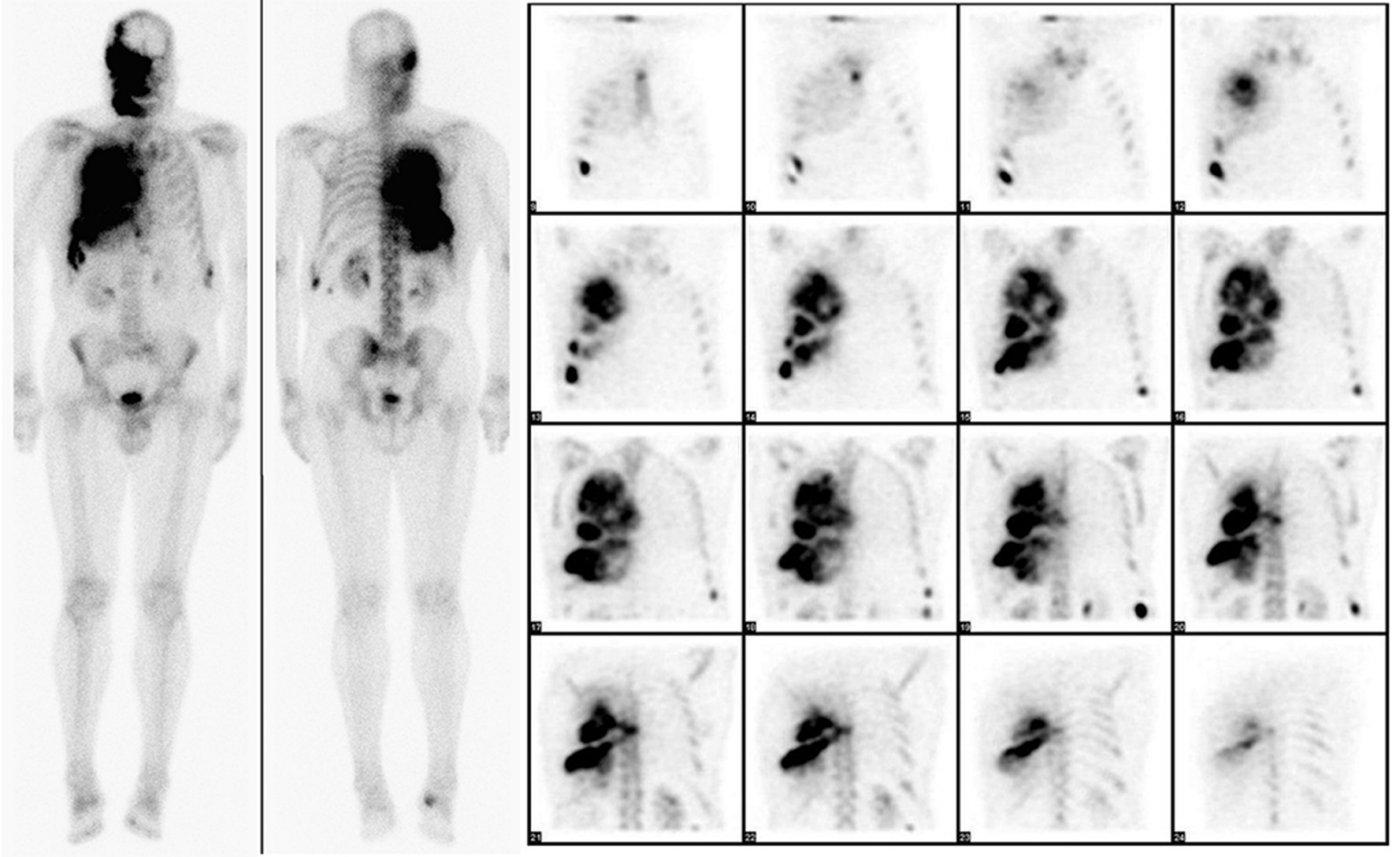
^99m^Tc-methylene diphosphonate whole-body bone scan showing intense uptake in several right facial bones, including the frontal, zygomatic and nasal bones, maxilla and mandible. There was also intense uptake involving the right ribcage. Whole-body planar anterior and posterior images and single-photon emission CT coronal images of the thorax are shown.

**Figure 2.**
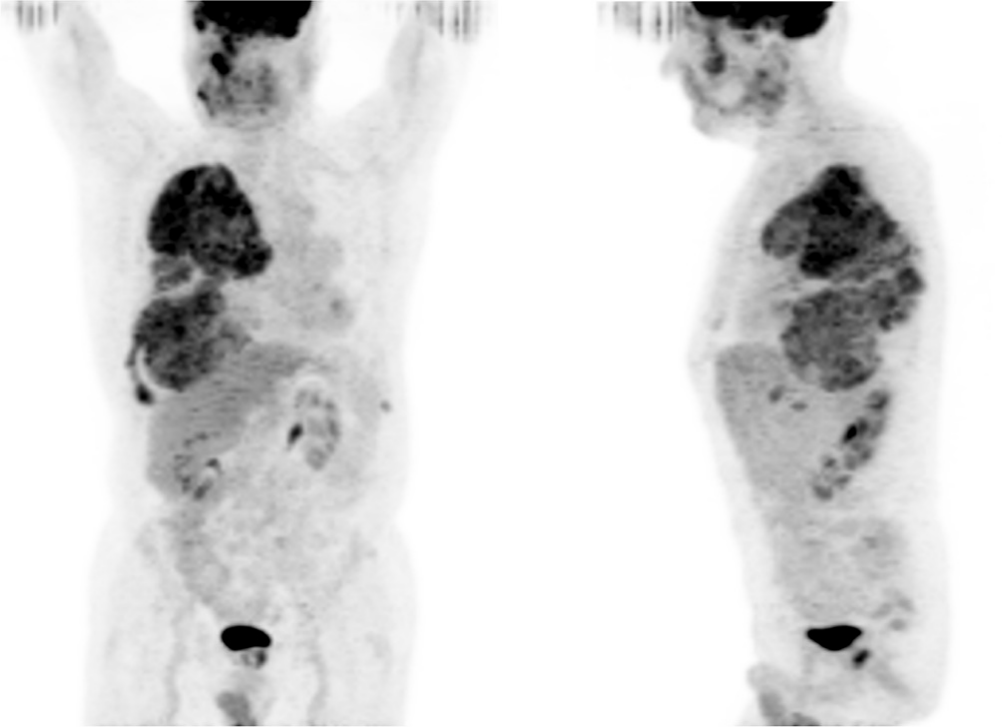
^18^F-fludeoxyglucose positron emission tomography/CT maximum intensity projection images showing intense heterogeneous ^18^F-fludeoxyglucose uptake in the bones of the right face and right hemithorax.

**Figure 3.**
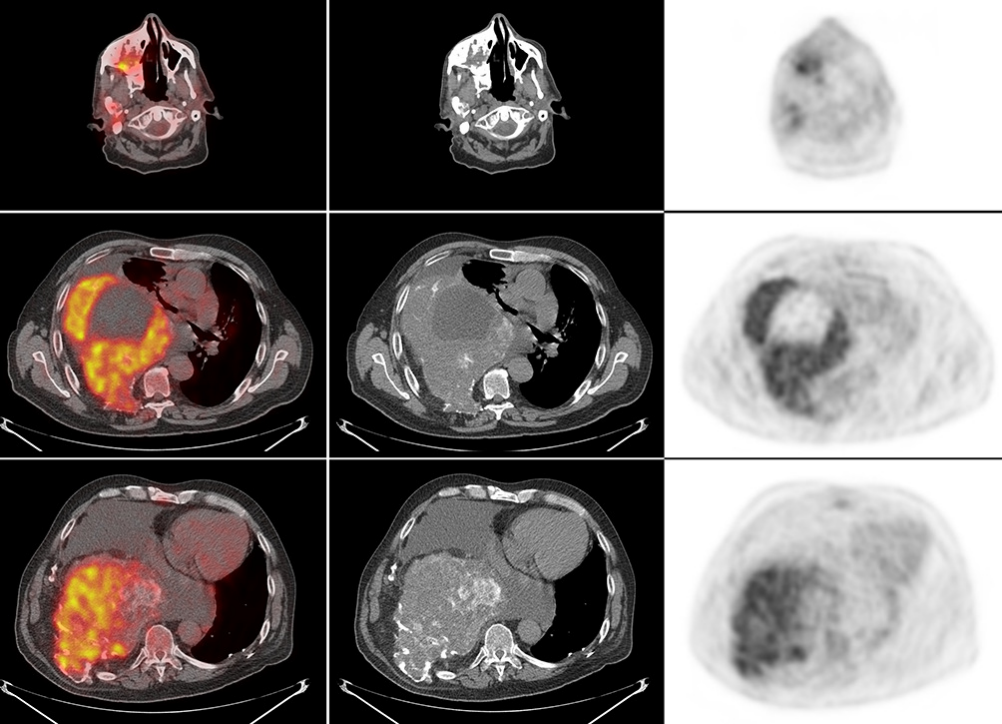
The ^18^F-fludeoxyglucose uptake in the facial bones was variable, with maximum standardized uptake value ranging from 2.1 to a maximum of 5.4 in the right maxillary bone. The right hemithorax lesions appeared to arise from the right eighth and ninth ribs, with involvement of the T7 vertebra, which was collapsed. The maximum standardized uptake value in the right hemithorax lesions ranged from 4.0 to 7.5.

**Figure 4.**
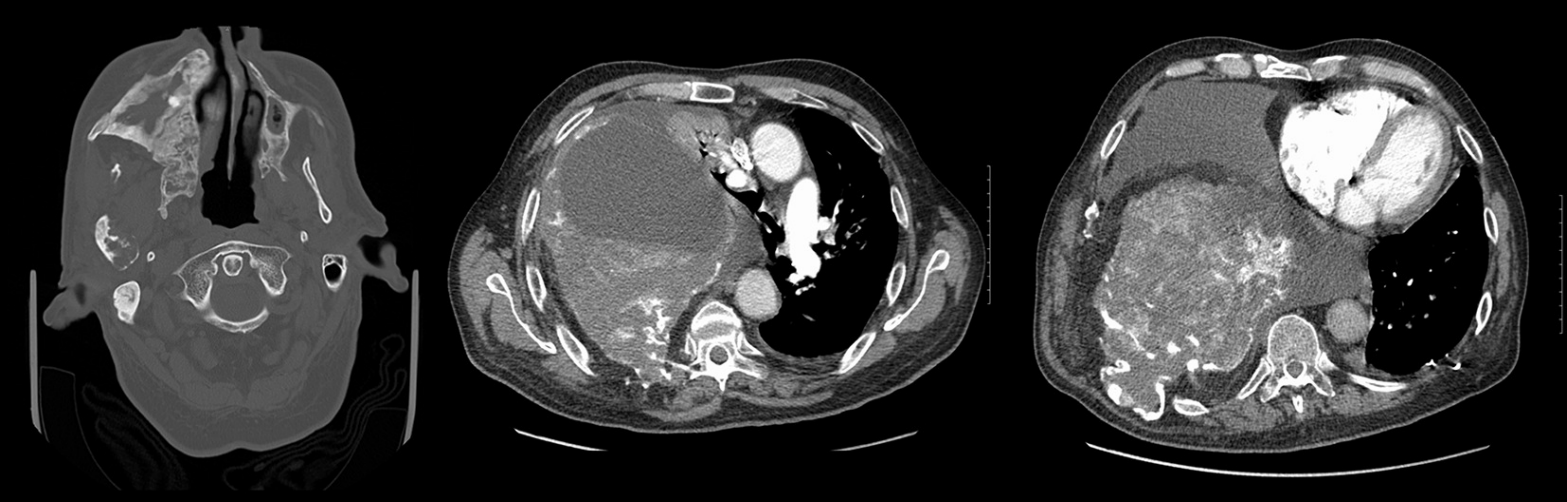
A CT scan performed 8 years later showed stability of the benign fibrous dysplasia lesions in the skull and thorax.

A 72-year-old male with a long-standing history of polyostotic fibrous dysplasia presented with chest and back pain and was evaluated with a ^99m^Tc-methylene diphosphonate (MDP) bone scan, which revealed intense uptake in several right facial bones, including the frontal, zygomatic and nasal bones, maxilla and the mandible. There was also intense uptake involving the right ribcage (Figure 1). The patient was referred for ^18^F-fludeoxyglucose (FDG) positron emission tomography (PET)/CT imaging to evaluate for possible malignant transformation. Maximum intensity projection images showed intense heterogeneous ^18^F-FDG uptake in the bones of the right face and right hemithorax (Figure 2). The ^18^F-FDG uptake in the facial bones was variable, with the maximum standardized uptake value (SUV_max_) ranging from 2.1 to a maximum of 5.4 in the right maxillary bone. The right hemithorax lesions appeared to arise from the right eighth and ninth ribs, with involvement of the T7 vertebra, which was collapsed. The SUV_max_ in the right hemithorax lesions ranged from 4.0 to 7.5 (Figure 3). Mildly FDG-avid lesions were also noted in T4 (SUV 3.1), left lateral tenth rib (SUV 2.2) and left sacral wing (SUV 2.4). The heterogeneous nature of ^18^F-FDG uptake and the wide range of SUV_max_ values raised concern of malignant transformation (or sarcomatous degeneration), and follow-up with a CT scan was recommended. Subsequent 8 years of follow-up with CT scans (Figure 4) did not reveal the development of any aggressive bone lesions, and the patient remains clinically stable with no evidence of malignant transformation.

## Discussion

Fibrous dysplasia accounts for approximately 7% of benign bone tumours and is a developmental disorder of unknown aetiology. Malignant transformation has been reported in 0.4% of all cases, although this rises to 4% in familial cases such as McCune–Albright.^1^ In most reported cases in the literature, the diagnosis was made during childhood and malignant transformation was diagnosed during the third or fourth decade of life. The most common malignant tumour types that are the result of malignant transformation are osteosarcoma (55%), fibrosarcoma (30%) and chondrosarcoma (15%). Craniofacial bones are the most common sites of malignant transformation, followed by the femur, tibia and pelvis.^1^

In older adults, fibrous dysplasia is associated with several complications, the most common of which are fractures. The highest fracture rate occurs in the 6- to 10-year-old age group and decreases into early adulthood, where it stabilizes for the rest of the lifespan at approximately 0.1 mean number of fractures per patient per year. Another complication encountered with fibrous dysplasia involving the spine is scoliosis. Untreated scoliosis can lead to pulmonary compromise and death; however, fixation is very effective for long-term stabilization.^2^

Variable ^18^F-FDG uptake in cases of fibrous dysplasia has been described, from no uptake^3^ to intense uptake, usually in cases where it mimics metastatic disease.^4–13^ ^18^F-FDG uptake can vary dramatically, even within the same patient, between two different PET/CT studies.^14^ The highest SUV_max_ reported in the literature for a benign fibrous dysplasia lesion was an SUV_max_ of 11.42, reported by Su et al.^15^

The use of ^18^F-FDG PET/CT scan in the evaluation of malignant transformation of fibrous dysplasia has been described in one case report by Berrebi et al,^16^ with the authors suggesting a possible role for ^18^F-FDG PET/CT scan. In this report, however, the authors failed to make a strong case for the usefulness of PET/CT scan as there was no premalignant transformation scan to compare with, to see if the PET/CT findings changed as a result of malignant transformation. In addition, the malignant transformed lesion had both hypometabolic and hypermetabolic regions, which made it impossible to characterize the lesion by PET/CT, and a diagnosis was only possible *via* biopsy.^16^ In another case by Santiago et al, an ^18^F-FDG-avid lesion was followed up for 1 year and did not demonstrate malignant degeneration.^17^

Studies in the literature that describe intense ^18^F-FDG uptake in monostotic or polyostotic fibrous dysplasia do not have long-term follow-up of their patients longer than 12 months.^4–13,17^ In our case, owing to the highly heterogeneous yet intense uptake on both the bone and ^18^F-FDG PET/CT scans, malignant transformation could neither be confirmed nor excluded. Long-term follow-up with CT scans over 8 years, however, confirmed the absence of malignant transformation. We suspect that ^18^F-FDG PET/CT scan is probably not useful in the evaluation of malignant transformation of fibrous dysplasia. Comparison of ^99m^Tc-MDP and ^18^F-FDG PET/CT images of fibrous dysplasia is also unlikely to be helpful, as significant mismatches between ^99m^Tc-MDP and ^18^F-FDG uptake have been described, with doubtful clinical significance.^18^ PET/CT scan may be useful in very specific applications in fibrous dysplasia, for example, to help guide potential biopsy sites by identifying the most ^18^F-FDG-avid bone lesions, or for long-term follow-up of non-^18^F-FDG-avid lesions or lesions with very low grade ^18^F-FDG avidity, to determine if there is development of new hypermetabolic lesions that would be suggestive of malignant transformation.

## Learning points

1. Benign fibrous dysplasia lesions can show intense radiotracer uptake on a ^99m^Tc-MDP bone scan as well as on ^18^F-FDG PET/CT scan.
2. SUV_max_ of fibrous dysplasia lesions on ^18^F-FDG PET/CT scan cannot differentiate between a benign fibrous dysplasia lesion and a fibrous dysplasia lesion that has undergone malignant transformation or sarcomatous degeneration.
3. ^18^F-FDG PET/CT scan may play a role in specific circumstances such as guiding a potential biopsy site by identifying the most ^18^F-FDG-avid bone lesions, or long-term follow-up of non-^18^F-FDG or very low-grade ^18^F-FDG-avid fibrous dysplasia lesions.
4. Long-term follow-up with anatomic diagnostic imaging such as CT or MRI may be necessary in most cases of fibrous dysplasia to ensure stability and exclude malignant transformation or sarcomatous degeneration.

## Consent

Informed consent to publish this case (incuding images and data) was obtained and is held on record.
